# Extracellular vesicles from canine mammary tumor cells promote macrophage M2 polarization and enhance tumor progression

**DOI:** 10.1007/s11259-026-11179-3

**Published:** 2026-03-30

**Authors:** Na-Kyoung Chi, Se-Hoon Kim, Ga-Hyun Lim, Ki-Hoon Song, Ju-Hyun An, Min-Ok Ryu, Kyoung-Won Seo

**Affiliations:** 1 Department of Veterinary Internal Medicine, College of Veterinary Medicine, Seoul National University, Seoul, Republic of Korea; 2 VIP animal medical center, Seoul, Republic of Korea; 3 Research Institute, ViroCure Inc., Seoul, Republic of Korea; 4 Department of Veterinary Emergency and Critical Care Medicine, College of Veterinary Medicine, Kangwon National University, Seoul, Republic of Korea

**Keywords:** Canine mammary tumor, Epithelial-mesenchymal transition (EMT), Extracellular vesicle, Tumor-associated macrophages (TAMs), Tumor microenvironment

## Abstract

Extracellular vesicles (EVs) derived from tumor cells play a crucial role in modulating and reprogramming the tumor microenvironment (TME), thereby promoting tumor progression. While previous studies have investigated the impact of tumor-derived EVs on macrophages in various cancers, such as bladder cancer and osteosarcoma, and in canines, particularly in melanoma, little is known about the effects of canine mammary tumors influence on macrophage polarization. This study aimed to elucidate the functional role of EVs derived from CIPp cells, a canine mammary tumor cell line, in macrophage polarization and tumor progression. CIPp-derived EVs were isolated and characterized, and their effects on macrophage phenotype were examined using the canine macrophage cell line DH82. Exposure to CIPp-EVs induced DH82 macrophages to adopt a tumor-associated macrophage (TAM)-like M2 phenotype, as confirmed by quantitative RT-PCR, immunofluorescence, and flow cytometry. CIPp-EV treatment significantly upregulated M2-associated markers (CD206, VEGF-A, IL-10, and COX2) while downregulating M1-associated markers (iNOS and IL-6). Furthermore, conditioned media (CM) from CIPp-EV–treated macrophages enhanced CIPp tumor cell migration and induced epithelial–mesenchymal transition (EMT), evidenced by the reduction of E-cadherin and increased expression of vimentin, fibronectin, and α-SMA. Together, these findings demonstrate that CIPp-derived EVs reprogram the TME by driving TAM-like M2 macrophage polarization, which in turn promotes tumor cell migration and EMT, thereby facilitating tumor progression and metastasis.

**Supplementary Information:**

The online version contains supplementary material available at 10.1007/s11259-026-11179-3.

## Introduction

In the evolving landscape of cancer biology, Tumor-derived extracellular vesicles (TDEs) have recently emerged as key regulators of tumor growth, metastasis, and immune evasion (Zhang and Yu 2019; Olejarz et al. 2020). TDEs are nanosized, lipid bilayer-enclosed vesicles that carry various bioactive molecules, including tumor-derived proteins, lipids, microRNAs, and mRNA (Whiteside 2016). These vesicles deliver molecular cargo to recipient cells within the tumor microenvironment (TME), reprogramming it into an immunosuppressive and pro-tumoral niche (Reale et al. 2022).

The TME is a complex and dynamic ecosystem composed of immune cells, fibroblasts, and endothelial cells, which interact closely with tumor cells (Yang et al. 2020a). Among the immune cells, macrophages play a central role in shaping the immune landscape under the influence of TDEs. Depending on external stimuli, macrophages can differentiate into either tumor-suppressive M1 or tumor-promoting M2 phenotypes (Boutilier and Elsawa 2021). In many solid tumors, TDEs have been shown to induce M2 polarization, leading to the formation of tumor-associated macrophages (TAMs) (Baig et al. 2020). TAMs secrete immunosuppressive cytokines (e.g., IL-10, TGF-β) and tumor-promoting factors (e.g., VEGF, MMPs), which suppress immune responses, promote angiogenesis, and induce epithelial–mesenchymal transition (EMT), thereby enhancing tumor cell motility and invasiveness (Noy and Pollard 2014; Chen et al. 2019). EMT is a key process in cancer metastasis and is strongly associated with poor prognosis (Ribatti et al. 2020).

Through these mechanisms, TDEs create a tumor-favorable microenvironment via the TDE–TAM axis (Chen et al. 2024). This axis has been extensively studied in various human solid tumors (Guo et al. 2020; Chen et al. 2021), including breast cancer, lung cancer (Pritchard et al. 2020), and glioblastoma (Gabrusiewicz et al. 2018). In breast cancer, TDEs have been reported to not only promote M2 polarization (Hao et al. 2023) but also enhance cancer cell migration and lymph node metastasis (Piao et al. 2018). Consequently, inhibitors of extracellular vesicle (EV) secretion are currently being explored as potential anti-cancer therapies (Li et al. 2022b; Zhang et al. 2020).

Despite significant advances in human oncology, studies on EV–immune cell interactions remain limited in veterinary oncology. Canine mammary tumors (CMTs), one of the most common neoplasms in intact female dogs (Sleeckx et al. 2011), share clinical and molecular similarities with human breast cancer, making them valuable comparative oncology models (Abdelmegeed and Mohammed 2018). Previous studies have demonstrated that EVs derived from the CMT cell line CIPp may enhance proliferation and migration of CIPp cells themselves (Moccia et al. 2023). However, their effects on immune cells—particularly on macrophage polarization—remain unclear. To date, research on EV–macrophage interactions in dogs has been limited to a few melanoma models (Kim et al. 2022), in which EVs were shown to promote M2 polarization and reinforce immunosuppressive conditions.

This study was designed to test the hypothesis that EVs derived from the canine mammary gland tumor cell line CIPp polarize canine macrophages toward a TAM-like M2 phenotype, thereby promoting tumor progression via enhanced migration and EMT. This study provides novel insights into the role of EVs in shaping the immune landscape of canine mammary tumors. Given the conserved nature of EV–TAM interactions across species, this work may inform the development of TAM-targeted therapies in both veterinary and human oncology.

## Materials and methods

### Cell culture and isolation of EV

The canine malignant mammary gland tumor cell line CIPp, established from a primary tumor, was obtained from the Department of Veterinary Clinicopathology at Seoul National University. Cells were cultured in RPMI-1640 medium (Thermo Fisher Scientific, Waltham, MA, USA; #11875093) supplemented with 10% fetal bovine serum (FBS; Thermo Fisher Scientific; #16000-044) and 1% penicillin-streptomycin (PS; Thermo Fisher Scientific; #15140122) at 37◦C in a humidified atmosphere containing 5% CO2.

For conditioned media collection, CIPp cells were seeded at 5000 cells/cm² in 10-layer Culture Chambers (BioPioneer Inc., San Diego, CA, USA) and cultured in RPMI with 10% FBS until 80% confluence. The cells were washed three times with phosphate-buffered saline (PBS, Welgene, Gyeongsan, Gyeongbuk, Republic of Korea; #LB 004 − 01) to remove residual FBS and incubated for 24 h in serum-free CD 293 medium (Thermo Fisher Scientific; #11913-019) supplemented with 1% GlutaMAX and 1% sodium pyruvate before harvesting the conditioned media (CM).

EVs were isolated from the CM using the tangential flow filtration (TFF)-based method. The conditioned media was first filtered through a 0.22-µm polyethersulfone membrane filter (Thermo Fisher Scientific) to remove cell debris and non-EV particles. The filtrate was then concentrated using a 700 kDa hollow-fiber cartridge (Cytiva, Marlborough, MA, USA) and diafiltered with PBS. The EV suspension was aliquoted into polypropylene disposable tubes, and stored at − 80 °C. Before use, frozen EVs were thawed completely at 4 °C. Characterization and profiling of EVs were performed in accordance with the Minimal Information for Studies of Extracellular Vesicles 2018 (Théry et al. 2018) recommended by the International Society of Extracellular Vesicles.

### Characterization of EV derived from CIPp cells

To verify that the isolated particles were EVs, western blotting was performed on CIPp cell lysates and EV preparations to assess the enrichment of EV-specific protein markers. Cells were lysed using a lysis buffer (Elpis Biotech, Daejeon, Republic of Korea; #EBA-1149) and kept on ice for 30 min. The lysates were then centrifuged at 12,000 rpm for 20 min at 4 °C to remove cell debris. Protein concentrations were determined using a BCA Protein Assay Kit (Thermo Fisher Scientific; #23227). Equal amounts of protein were resolved by SDS–polyacrylamide gel electrophoresis and transferred to polyvinylidene fluoride (PVDF) membranes (MilliporeSigma, Darmstadt, Germany; #IPVH00010).

Membranes were blocked for 1 h at 21 °C in TBST buffer containing either 5% skim milk (LPS Solution, Daejeon, Republic of Korea; #SKI500) or 5% bovine serum albumin (BSA, GeorgiaChem, Hwaseong, Republic of Korea; #G.C-BS1005). After blocking, the membranes were incubated overnight at 4 °C with primary antibodies (1:1,000). Specifically, antibodies against CD9 (Cell Signaling Technology, Danvers, MA, USA; #13174), CD81 (System Biosciences, Palo Alto, CA, USA; #EXOAB-CD81A-1) and Alix (Cell Signaling Technology; #2171) were used as EV-positive markers. Additionally, β-actin (Cell Signaling Technology; #4970) was employed as a negative marker to assess the depletion of cellular contaminants.

Following TBST washes, the membranes were incubated for 1 h at 21 °C with HRP-conjugated secondary antibodies (1:2,000), including anti-rabbit and anti-mouse antibodies (ABclonal Technology; #AS014, #AS003). Protein bands were visualized using an enhanced chemiluminescence detection reagent (SmartGene, Seongnam, Republic of Korea; #SG-PR-HECL), and band images were acquired using the LAS-4000 imaging system (GE Healthcare Biosciences, Milwaukee, WI, USA).

Nanoparticle tracking analysis (NTA) was performed to assess the size and concentration of EV samples, using a 642-nm laser NanoSight LM10 (Malvern Panalytical, Amesbury, UK). EV samples were diluted in PBS and analyzed using NTA 3.2 software (Malvern Panalytical Ltd., Worcestershire, UK). For each sample, at least five videos were recorded and averaged to obtain representative measurements.

EV morphology was further evaluated by transmission electron microscopy (TEM). A 10 µl EV suspension was applied on a carbon-coated grid and allowed to absorb for 1 min, followed by removal of excess liquid with filter paper. A 2% uranyl acetate solution was then applied for 20 s as a negative stain, and excess stain was gently blotted before imaging. Images were acquired using a JEM1010 transmission electron microscope (Jeol Ltd., Tokyo, Japan) operating at 80 kV. All procedures were performed in triplicate to ensure reproducibility.

### DH82 cell culture and EV treatment

The canine macrophage cell line DH82 was purchased from ATCC (ATCC number: CRL-10389, Manassas, VA, USA). Cells were cultured in Dulbecco’s Modified Eagle Medium (DMEM, Solbio, Suwon, Republic of Korea, #DME-001) supplemented with 10% FBS and 1% PS, maintained at 37 °C in a 5% CO₂ incubator. DH82 cells were seeded for 12 h, followed by a media change and treated with CIPp-EVs for an additional 36 h.

### Cell viability assay

DH82 cell viability following exposure to CIPp-EVs was assessed using the Cell Counting Kit-8 (CCK-8, Dojindo Laboratories, Kumamoto, Japan, #CK04), following the manufacturer’s protocol. DH82 cells were seeded in 96-well plates at a density of 1.5 × 10³ cells/well and cultured overnight to allow attachment. The medium was then replaced, and cells were treated with either fresh medium alone, 20% PBS, 10% EVs (19.5 µg/ml), or 20% EVs (39 µg/ml) for 36 h. After adding 10 µl of CCK solution and incubating for 2 h, cell viability was measured using Epoch Microplate Spectrophotometer (BioTek Korea Co., Ltd., Seoul, Republic of Korea).

### Morphological observation

DH82 cells were seeded at a density of 1 × 10^5^ cells/well in 6-well plates for 12 h. Seeded DH82 cells were treated with CIPp-EVs (39 µg/ml) for 36 h. Cell morphology was observed using a Leica DMil microscope (Leica Microsystems, Wetzlar, Germany) to assess EV-induced morphological changes.

### Quantitative real-time PCR (qRT-PCR) of DH82 cells

DH82 cells were seeded at 2 × 10⁵ cells/well in 6-well plates and allowed to adhere for 12 h before being treated with CIPp-EVs (39 µg/ml) for 36 h. After treatment, total RNA was extracted using Easy-Blue Total RNA Extraction Kit (Intron Biotechnology, Daejeon, Korea, #17061) according to the manufacturer’s instructions, and RNA purity and concentration were measured using a spectrophotometer (NanoPhotometer NP80, Implen GmbH, Munich, Germany). Quantitative PCR was performed on a Qunatstudio 1 (Applied Biosystems, CA, USA) using SYBR Green qPCR Master Mix (SmartGene, #SG-SYBR-ROXH). Target gene expression levels were normalized to GAPDH, which served as the housekeeping control. Primer sequences utilized in this study are provided in Table S1. Triplicate reactions were carried out for all samples.

### Immunofluorescence assay

To analyze M1/M2 surface marker expression in DH82 cells, an immunofluorescence assay was performed. DH82 cells were seeded at a density of 2 × 10⁴ cells per confocal dish (SPL Life Sciences, Pocheon, Gyeonggi-do, Republic of Korea, #101350) and incubated for 12 h. Cells were then treated with CIPp-EVs (39 µg/ml) for 36 h. As a positive control for M1 polarization, cells were exposed to 100 ng/ml Lipopolysaccharide (LPS, Sigma-Aldrich, St. Louis, MO, USA, #L4391) for the same duration. Following treatment, cells were washed with PBS and fixed with 4% paraformaldehyde for 15 min at 21 °C. After additional PBS washes, cells were blocked with 5% BSA for 30 min at 21 °C to prevent nonspecific binding. FITC-conjugated anti-mouse CD206 antibody (1:100 dilution, BioLegend, San Diego, CA, USA, #141703) and APC-conjugated anti-mouse CD86 antibody (1:10 dilution, Miltenyi Biotec, Bergisch Gladbach, Germany, #130-102-558) were applied to the cells, followed by incubation for 1 h at 21 °C. Cells were then washed three times with PBS, and coverslips were mounted with DAPI-containing mounting solution (Thermo Fisher Scientific, #00-4959-52). Fluorescence images were acquired using an EVOS FL imaging system (Thermo Fisher Scientific).

### Flow cytometry analysis

Flow cytometric analysis was performed to evaluate M1 and M2 surface marker expression in DH82 cells. Cells were seeded at a density of 2 × 10⁵ cells per well in 6‑well plates and allowed to adhere for 12 h. The cultures were then divided into three groups: untreated controls, LPS-treated cells (200 ng/mL, 36 h) to induce M1 polarization, and CIPp-EV-treated cells (39 µg/mL, 36 h) to assess EV-mediated modulation of macrophage phenotype. Following treatment, cells were collected, washed with PBS, and fixed in 4% paraformaldehyde for 15 min at 21 °C. To reduce nonspecific binding, cells were blocked with 5% BSA for 30 min. Staining was performed using the same FITC-conjugated CD206 and APC-conjugated CD86 antibodies described in the immunofluorescence assay, using identical dilutions. After a 1 h incubation at 21 °C in the dark, the cells were washed with PBS and resuspended in 200 µL PBS to obtain a single-cell suspension. Samples were analyzed using a FACS Aria II flow cytometer (BD Biosciences, San Jose, CA, USA), and data were processed with FlowJo software (Tree Star, Ashland, OR, USA).

### Conditioned media preparation

DH82 cells were seeded in 100 mm dishes at a density of 1 × 10^6^ cells in complete DMEM medium for 12 h. After attachment, the cultures were divided into three groups. The control group was maintained in fresh complete DMEM without EVs for an additional 36 h. For EV treatment, cells were exposed to CIPp-EVs at 20% (v/v) for either 24–36 h. Following the respective incubation periods, the cells were washed twice with PBS to completely remove any residual CIPp-EVs. Subsequently, the medium was replaced with fresh DMEM medium with 10% Exo-free FBS (Thermo Fisher Scientific, #A2720803) and 1% PS, and the cells were cultured for an additional 24 h to produce CM. The CM was harvested, centrifuged at 800 x g for 3 min to remove residual cells, and the supernatant was stored at − 80 °C until use.

### Wound healing assay

CIPp cells were seeded in 12-well plates at a density of 1 × 10^5^ cells/well and cultured until confluence. To inhibit proliferation, cells were treated with 2 µg/ml of mitomycin (Enzo Life Science, Farmingdale, NY, USA, #BML-GR311) for 2 h. The cell monolayer was then scratched with a 1 ml pipette tip and cell debris was removed by washing twice with PBS. The remaining adherent cells were cultured in complete DMEM supplemented with 50% CM. Wound healing was monitored at 0, 3, and 6 h under a microscope using a TCapture program (Tucsen Photonics, Fuzhou, Fujian, China). Wound width was quantified using the ImageJ software (National Institutes of Health, Bethesda, MD, USA) and relative width closure (%) was calculated using the following formula: [(Relative width at 0 h – relative width at 3–6 h)/relative width at 0 h] ×100.

### qRT-PCR of CIPp cells

CIPp cells were seeded at a density of 2 × 10^5^ cells/well in 6-well plates and cultured for 12 h. Seeded CIPp cells were then treated with 50% complete DMEM and 50% CM for 48 h. Following incubation, total RNA was extracted and EMT marker expression was analyzed by qRT-PCR using the same procedure described for DH82 cells.

### Statistical Analysis

All data were analyzed using the GraphPad Prism software (version 10.1.2, GraphPad Software Inc., San Diego, CA, USA). For the CCK-8 cell viability assay, data were analyzed using one-way ANOVA with Tukey’s multiple comparison test to assess differences among groups. For experiments involving comparisons between two groups (e.g., qRT-PCR, flow cytometry and wound healing), unpaired two-tailed Student’s t-tests were used. Data are presented as the mean ± SD, and *p* < 0.05 was considered statistically significant.

## Results

### Characterization of CIPp-EVs

**Fig. 1:**
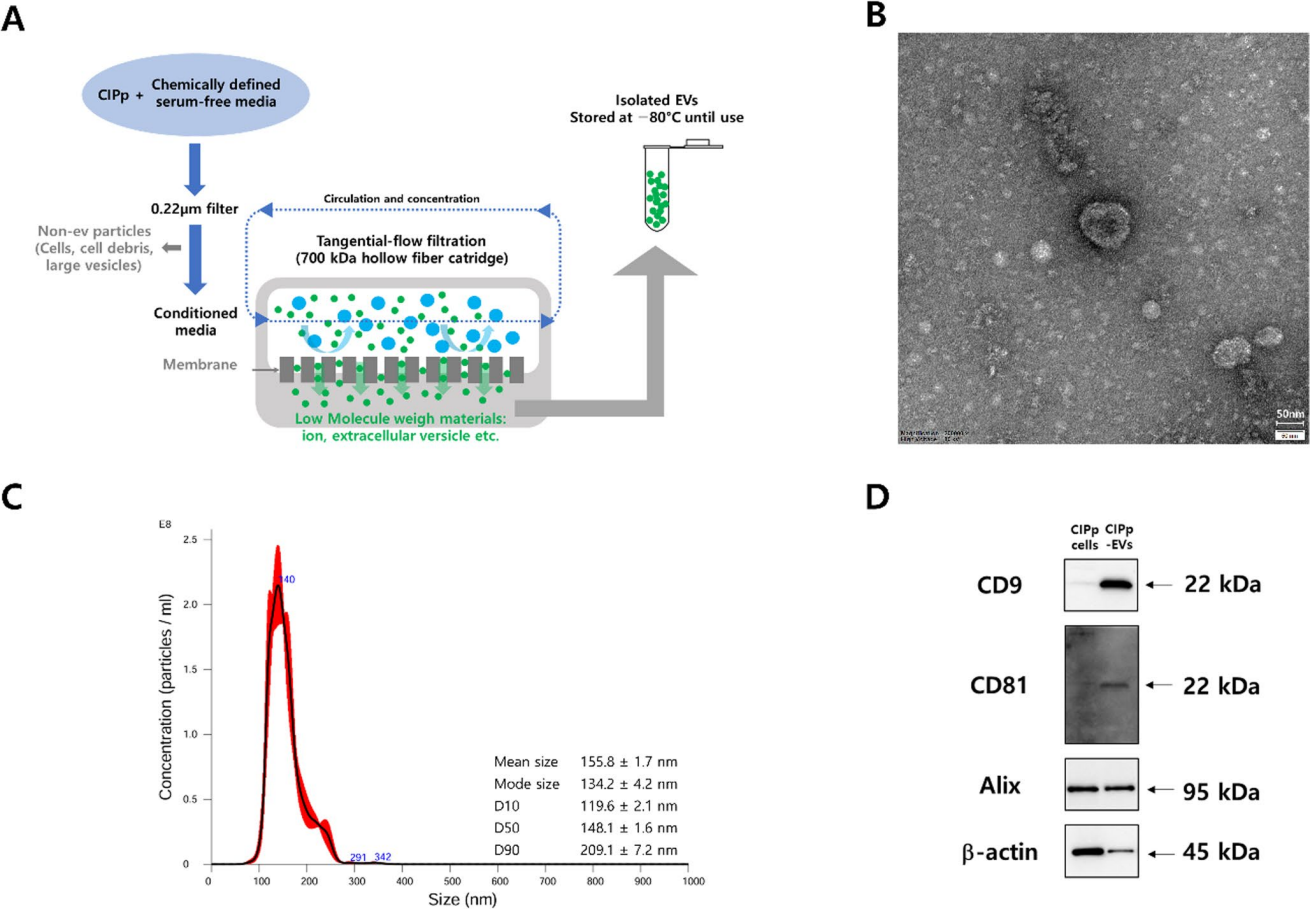
Characterization of EVs derived from CIPp cells. (**A**) Schematic overview of EV isolation using TFF. (**B**) TEM image showing the typical round morphology of CIPp-EVs. Scale bar: 50 nm. (**C**) Representative NTA histogram demonstrating the size distribution of CIPp-EVs, with a mean diameter of approximately 140 nm. (**D**) Western blot showing selective enrichment of EV-associated markers (CD9 and CD81) in CIPp-EVs relative to parental cell lysates

EVs were isolated from CIPp cells using TFF and the overall isolation process was schematically illustrated (Fig. 1A). TEM confirmed that the isolated EVs displayed a typical round morphology with an approximate diameter of 50 nm (Fig. 1B). As expected from the distinct measurement principles, NTA showed that the size distribution of CIPp-EVs was centered around 140 nm (Fig. 1C). Western blotting confirmed the enrichment of EV markers CD9 and CD81 in CIPp-EVs compared with CIPp cells (Fig. 1D). Taken together, these findings verify that TFF-based isolation effectively yields structurally intact EVs with uniform characteristics, supporting their suitability for downstream applications.

### Impact of CIPp-EVs on macrophage viability and morphology

**Fig. 2:**
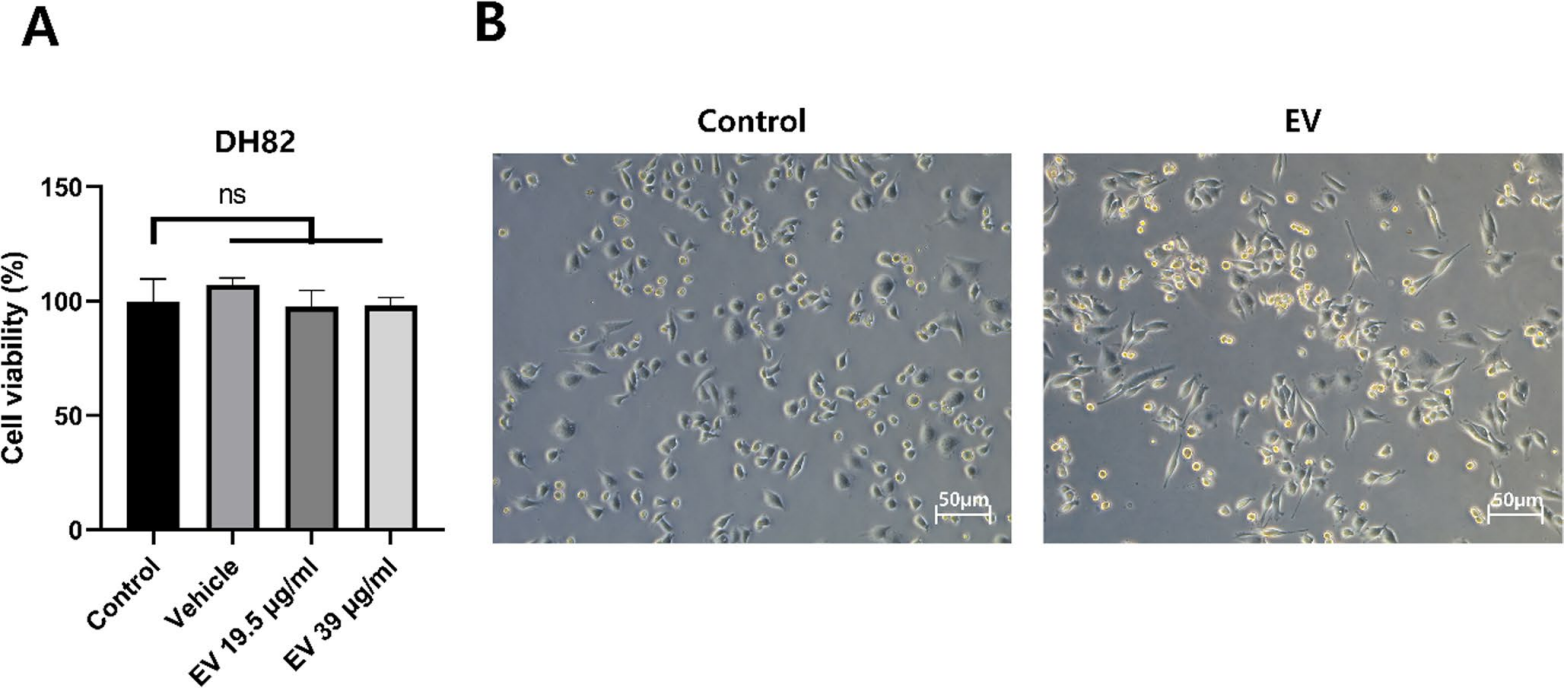
Effects of CIPp-EVs on Canine Macrophages. (**A**) Viability of DH82 cells after 36 h of exposure to CIPp-EVs (19.5 and 39 µg/mL), assessed using the CCK-8 assay. No significant differences were detected compared with untreated controls. Data are presented as mean ± SD; statistical analysis was performed using one-way ANOVA. (**B**) Morphological changes in DH82 cells following 36 h of treatment with or without CIPp-EVs. EV-treated cells exhibited a noticeably elongated, spindle-like morphology compared with control cells. Images were acquired at 100× magnification

The effect of CIPp-EVs on the viability of DH82 cells was evaluated using a CCK-8 assay. Cells were exposed to two concentrations of CIPp-EVs (19.5 and 39 µg/ml) for 36 h. No significant difference in cell viability was observed between the control and EV-treated groups (Fig. 2A), indicating that CIPp-EVs did not significantly affect cell viability at either dose. Based on these results, the higher concentration (39 µg/ml) was selected for subsequent experiments.

In addition to evaluating cell viability, morphological changes in DH82 cells were examined after 36 h of treatment with or without CIPp-EVs. The EV-treated cells exhibited a more elongated shape compared to the control group, suggesting alterations in cell morphology and a shift toward an M2-like phenotype in response to EV exposure (Fig. 2B). This observation is consistent with previous studies (McWhorter et al. 2013; Heinrich et al. 2017; Pe et al. 2022), which showed that M2-polarized macrophages characteristically adopt an elongated morphology, in contrast to the rounded form of M1 or unstimulated M0 macrophages.

### CIPp-EVs promote M2 macrophage polarization

To investigate the effects of CIPp-EVs on macrophage polarization, DH82 cells were co-cultured with CIPp-EVs (39 µg/ml) for 36 h. The expression of both M1 and M2 macrophage markers was analyzed using qRT-PCR, immunofluorescence, and flow cytometry.

**Fig. 3:**
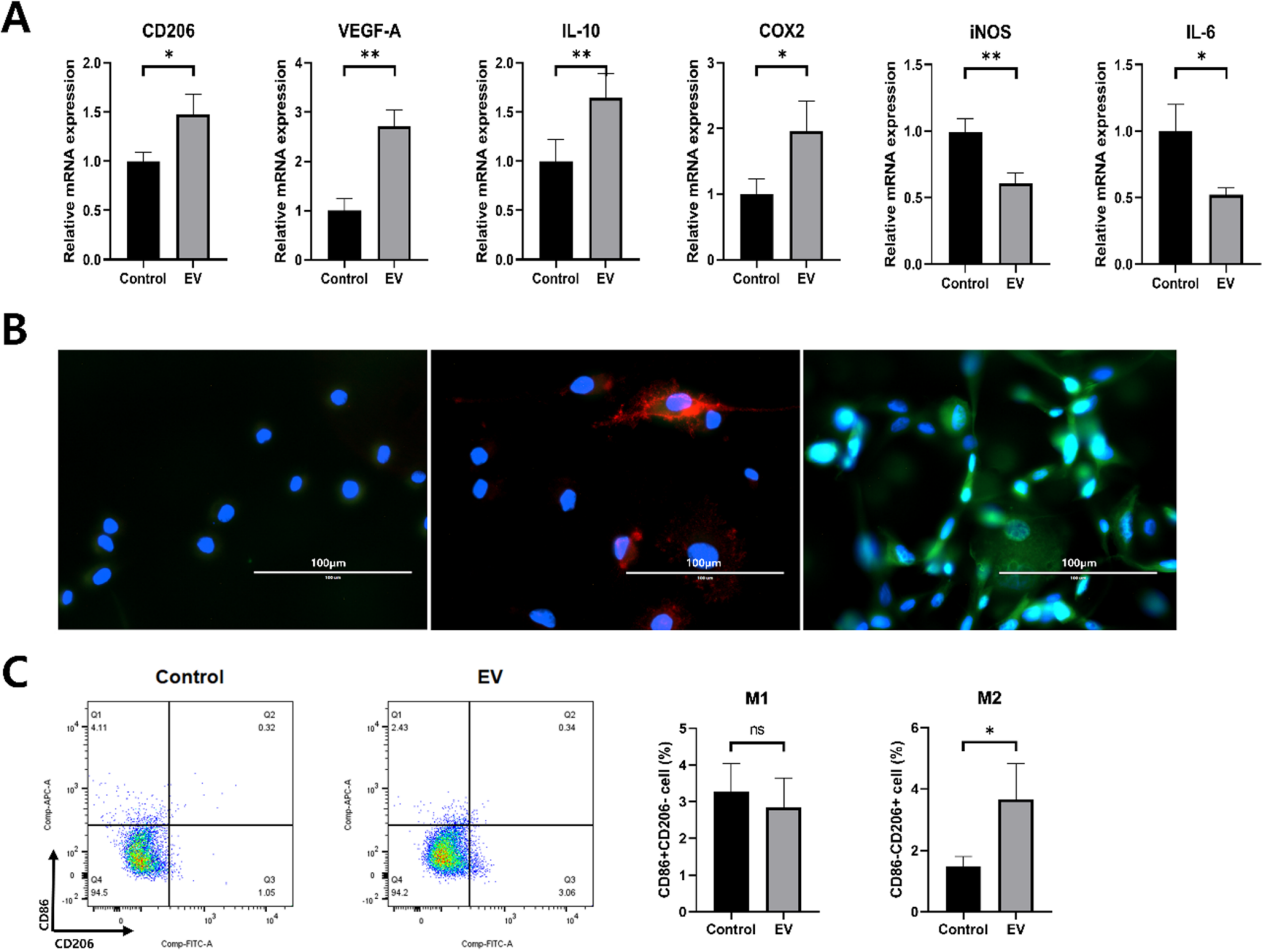
Effects of CIPp-EVs on macrophage polarization. DH82 cells were incubated with CIPp-EVs (39 µg/mL) for 36 h to evaluate EV-mediated polarization toward a tumor-associated macrophage (TAM)-like phenotype. (**A**) Relative mRNA expression of M1- and M2-associated cytokines was quantified by qRT-PCR. EV-treated cells showed increased expression of M2 markers (CD206, VEGF-A, IL-10, and COX2) and reduced expression of M1 markers (iNOS and IL-6) compared with untreated controls. *p < 0.05, **p < 0.01 versus control. (**B**) Immunofluorescence staining of CD86⁺ (M1, red) and CD206⁺ (M2, green) cells. EV treatment increased the proportion of CD206⁺ cells. Scale bar: 100 μm. (**C**) Surface expression of CD86^+^/CD206^−^ (M1) and CD86^−^/CD206^+^ (M2) macrophages assessed by flow cytometry. Data are presented as mean ± SD. *p < 0.05 by unpaired t-test analysis

qRT-PCR analysis showed significant upregulation of M2-associated genes (CD206, VEGF-A, IL-10, and COX2) and downregulation of M1-associated genes (iNOS and IL-6) in EV-treated macrophages compared with controls (Fig. 3A). These transcriptional changes are consistent with a shift toward an M2 phenotype. Immunofluorescence staining further supported this finding. Control cells exhibited minimal expression of either marker, while LPS-treated cells (positive control) displayed bright CD86 (red) staining characteristic of M1 polarization. By contrast, CIPp-EV–treated macrophages showed a pronounced increase in CD206 (green) signal, indicating M2 polarization at the protein level (Fig. 3B). Additionally, flow cytometry was performed to quantify the populations of M1 and M2 macrophages by measuring the surface expression of CD86 and CD206. The percentage of CD86^+^/CD206^−^ (M1) macrophages did not differ significantly between control and CIPp-EV-treated groups. However, CIPp-EV-treated cells showed a significant increase in CD86^−^/CD206^+^ (M2) macrophages (*p* < 0.05), confirming the polarization towards the M2, TAM-like phenotype (Fig. 3C).

Taken together, these findings demonstrate that CIPp-EVs effectively induce TAM-like M2 macrophage polarization, as indicated by the upregulation of M2 markers at both the gene and protein levels, and the increase in M2 macrophage populations. This shift towards the M2 phenotype may contribute to the immunosuppressive and tumor promoting properties observed in the tumor microenvironment.

### TAMs induced by CIPp-EVs enhance CIPp tumor progression

**Fig. 4:**
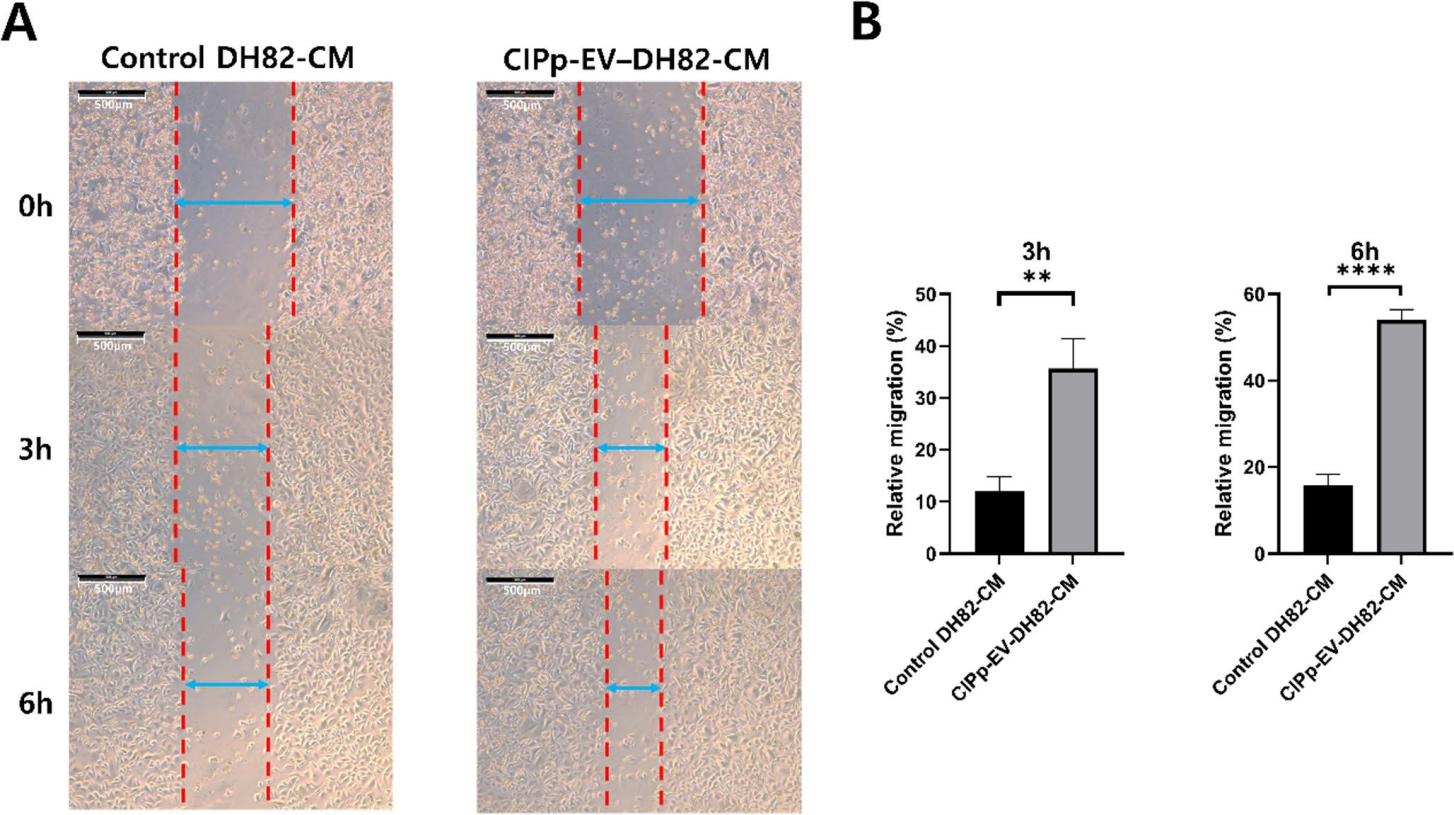
Effects of TAM-CM on the migratory activity of CIPp cells. CIPp cells were cultured in complete DMEM supplemented with 50% CM derived from DH82 macrophages. CM obtained from untreated DH82 macrophages is referred to as Control DH82-CM, whereas CM collected from DH82 macrophages treated with CIPp-EVs for 24 h to induce a TAM-like phenotype is referred to as CIPp-EV–DH82-CM. All experiments were performed in triplicate and repeated as three independent biological experiments. (**A**) Representative images of wound closure at 0, 3, and 6 h following treatment. Scale bar: 500 μm. (**B**) Quantification of relative migration rates. Data are presented as mean ± SD (*n* = 3). **p < 0.01, ****p < 0.0001 versus control

Having confirmed that CIPp-EVs induce TAM-like M2 macrophage polarization in DH82 cells (Fig. 3), the impact of these macrophages on CIPp tumor cell behavior was subsequently evaluated. Treatment of CIPp tumor cells with CM derived from DH82 macrophages exposed to CIPp-EVs for 24 h (CIPp-EV–DH82-CM) significantly increased the migratory activity of CIPp cells compared to CM from untreated DH82 macrophages (Control DH82-CM) (Fig. 4A). In contrast, CM collected from 36-hour EV-treated DH82 cells showed no significant effect (Fig. S1). These findings suggest that 24-hour CM contains a higher level of TAM-associated factors, and was therefore selected for all subsequent experiments.

**Fig. 5:**
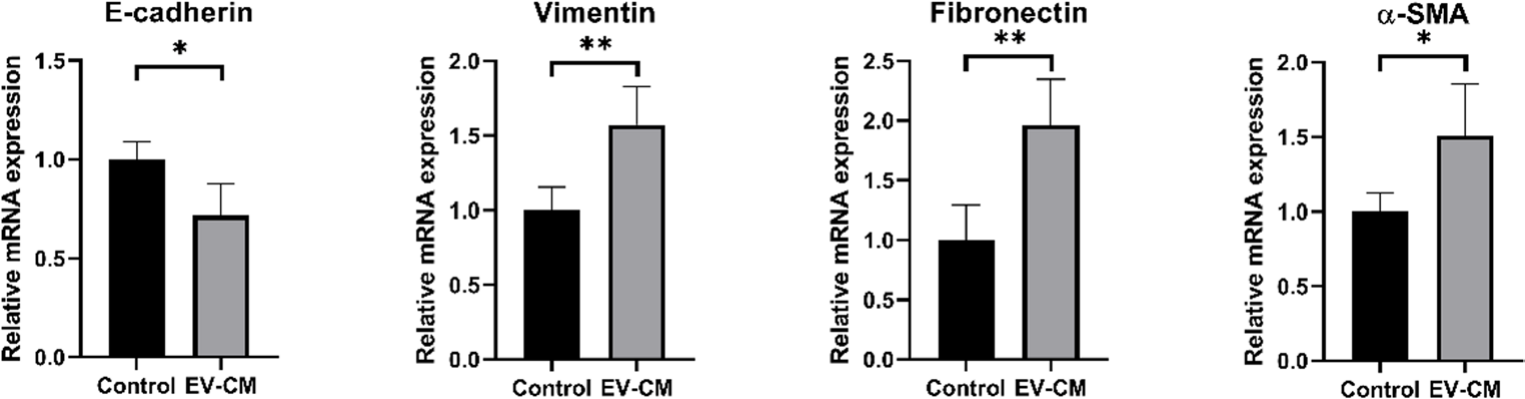
TAM-derived factors promote EMT in CIPp tumor cells. CIPp cells were treated for 48 h with CM derived from M2 polarized macrophages. qRT-PCR revealed downregulation of the epithelial marker (E-cadherin) and upregulation of mesenchymal markers (vimentin, fibronectin, and α-SMA), consistent with EMT induction. Data are presented as mean ± SD from three independent experiments. *p < 0.05, **p < 0.01 versus control group

Representative images taken at 0, 3, and 6 h illustrated a pronounced reduction in the wound gap in the CIPp-EV–DH82-CM group compared to the Control DH82-CM group. Quantitative analysis confirmed a significant increase in relative migration at 3 h (*p* < 0.01) and an even greater effect at 6 h (*p* < 0.0001) (Fig. 4B). To further assess TAM-mediated effects on tumor progression, EMT-related markers were analyzed. The CIPp-EV–DH82-CM group exhibited marked downregulation of the epithelial marker E-cadherin (*p* < 0.05) and upregulation of mesenchymal markers such as vimentin, fibronectin, and α-SMA (*p* < 0.01 for vimentin and fibronectin; *p* < 0.05 for α-SMA) (Fig. 5). These results indicate that TAM-derived factors promote EMT and enhance tumor invasiveness. Collectively, the results demonstrate that CIPp-EVs induce TAM-like polarization in macrophages, which in turn promotes CIPp tumor cell migration and EMT, thereby contributing to a more aggressive tumor microenvironment.

## Discussions

**Fig. 6:**
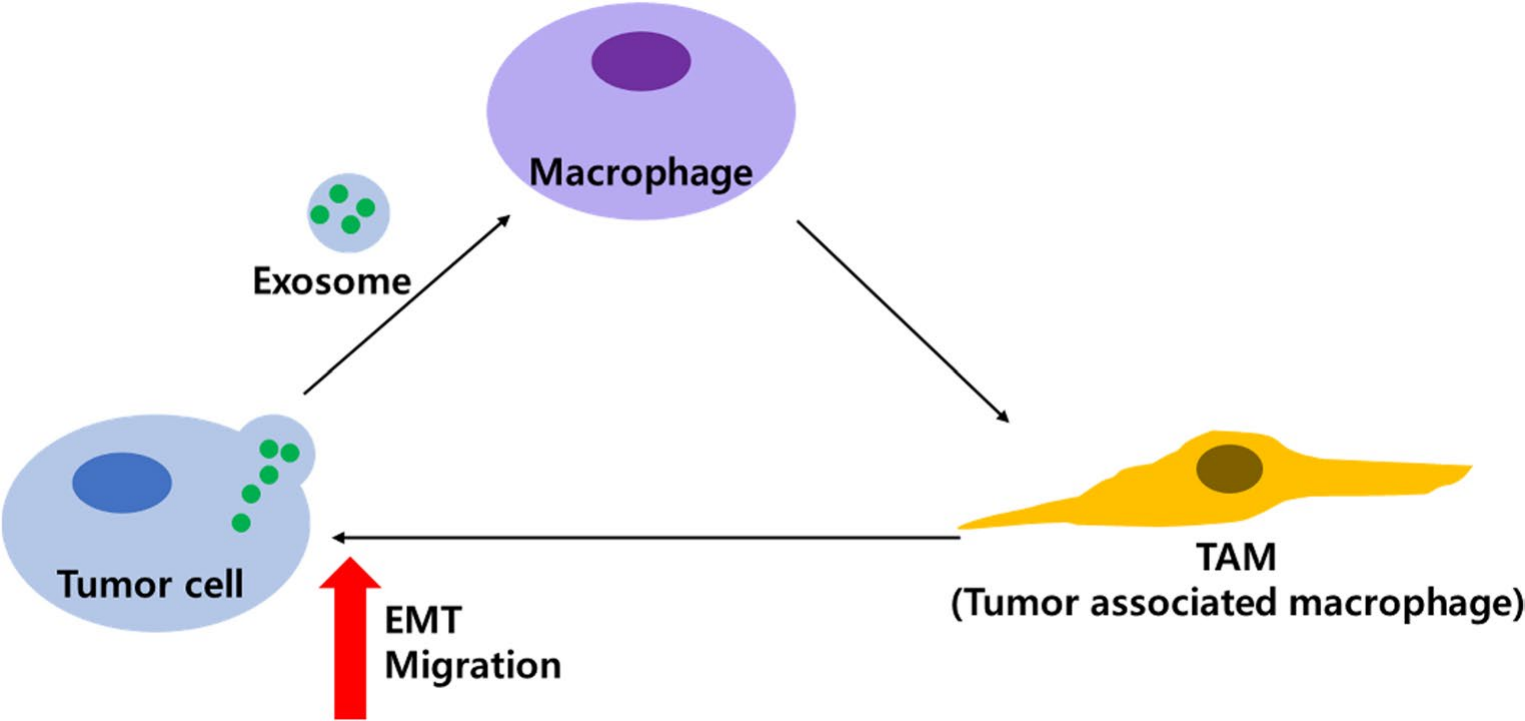
Schematic representation of the role of CIPp-EVs in tumor microenvironment remodeling and metastasis promotion. CIPp-EVs activate macrophages into TAMs, which secrete M2-associated cytokines, including IL-10, COX2, and VEGF-A. These cytokines contribute to an immunosuppressive environment and enhance tumor cell behavior. TAMs also promote metastatic potential by inducing EMT, characterized by increased tumor cell migration and invasiveness. This feedback loop between tumor cells and TAMs facilitates tumor progression and metastasis

This study demonstrates that CIPp-EVs promote M2 polarization of canine macrophage cell line DH82, and that these M2-polarized macrophages, in turn, enhance EMT and migratory potential in tumor cells. Together, these findings suggest that interactions among EVs, macrophages, and tumor cells establish a self-reinforcing feedback loop within the TME, thereby driving tumor malignancy and progression (Fig. 6).

TDEs are recognized as key modulators of the TME, notably by polarizing macrophages toward an M2 TAM phenotype (Baig et al. 2020; Wang et al. 2024a). In both canine and human mammary carcinomas, studies show that infiltration of M2 TAMs is strongly associated with advanced clinical stage, shorter survival, and disrupted extracellular matrix organization (Monteiro et al. 2018; Jeong et al. 2019; Allison et al. 2022; Garcia et al. 2025). TAMs have been shown to promote tumor progression through multiple mechanisms, including the secretion of immunosuppressive cytokines, angiogenic factors, and extracellular matrix–remodeling enzymes, thereby fostering an invasive and immune-suppressive microenvironment (Yang et al. 2020b; Bied et al. 2023). Beyond these correlative findings, mechanistic studies in human oncology have demonstrated that tumor-derived EVs act upstream of macrophage polarization toward the M2 phenotype, thereby promoting the establishment of a tumor-supportive microenvironment and enhancing tumor progression (Chen et al. 2020; Reed et al. 2021; Tian et al. 2023; Wang et al. 2024a). However, whether a comparable EV–TAM axis exists in canine tumors remains largely unexplored. The present study addresses this gap by providing functional evidence for EV-driven TAM polarization and its downstream effects in a canine mammary tumor model.

Previous studies have shown that the activation of the STAT3 signaling pathway reprograms macrophages toward a tumor-promoting M2-like phenotype across multiple cancers. (Takaishi et al. 2010; Fu et al. 2017; Ham et al. 2018; Mu et al. 2018; Irey et al. 2019; Mohammad et al. 2025). In our study, CIPp-EVs induced hallmark features of M2 polarization, including elongated morphology, increased CD206 expression, and elevated secretion of cytokines such as VEGF-A and IL-10 (McWhorter et al. 2013; Rőszer 2015; Wang et al. 2024b). Given that IL-10 is a known upstream activator of STAT3 in macrophages (Murray 2006; Hutchins et al. 2013), it is hypothesized that CIPp-EVs may engage a positive feedback mechanism wherein EV-induced IL-10 secretion reinforces STAT3 signaling, thereby stabilizing the TAM-like phenotype. While the direct activation of this signaling pathway was not experimentally validated in the current study, the observed pattern of IL-10 upregulation provides a strong rationale for future investigations into STAT3-dependent mechanisms for conditioning and the establishment of a protumorigenic microenvironment.

Importantly, M2-polarized macrophages are not only immunosuppressive but also potent inducers of EMT via secretion of IL-10, VEGF, and TGF-β (Feng et al. 2018; Li et al. 2022a; Zhang et al. 2024; Zhao et al. 2024). These cytokines repress epithelial markers such as E-cadherin while inducing mesenchymal markers including vimentin, N-cadherin, and fibronectin, thereby enhancing tumor cell motility and invasiveness (Ribatti et al. 2020). Multiple studies have demonstrated that IL-10, VEGF, and TGF-β secreted by TAMs are critical mediators of EMT, and inhibition of each factor effectively attenuates TAM-induced EMT and tumor cell invasiveness (Liu et al. 2013; Feng et al. 2018; Cai et al. 2019). In this study, CM from CIPp-EV–treated macrophages downregulated E-cadherin and upregulated vimentin and fibronectin in CIPp tumor cells, accompanied by enhanced migratory capacity, confirming EMT induction. It should be noted that the term “TAM-like macrophages” used in this study is based on marker expression patterns and in vitro functional effects, rather than comprehensive in vivo functional validation. Nevertheless, the collective findings suggest that CIPp-EVs reprogram macrophages toward TAM-like phenotype, potentially via a STAT3–IL-10 pathway, and these reprogrammed macrophages, in turn, drive EMT through IL-10 and VEGF signaling—establishing a self-reinforcing EV–TAM–tumor axis that promotes tumor progression in canine mammary carcinoma.

In this study, to ensure the high purity and integrity of EVs required for functional assays, tangential Flow Filtration (TFF) was utilized. This method allows for the scalable isolation of EVs with high yield and purity even from large-volume or diluted samples (Busatto et al. 2018; Liangsupree et al. 2021). Subsequent NTA analysis demonstrated that TFF-isolated EVs exhibited a narrow and uniform size distribution, supporting the suitability of this method for elucidating the biological roles of CIPp-EVs.

Given the central role of the EV–TAM axis in establishing a tumor-supportive microenvironment, targeting this pathway has emerged as a promising therapeutic strategy in preclinical cancer models (Im et al. 2019; Jiang et al. 2021; Li et al. 2022b; Peng et al. 2022). Our findings demonstrate that TDEs are key drivers of macrophage polarization. Therefore, strategies aimed at inhibiting EV biogenesis or secretion could effectively disrupt this pro-tumorigenic crosstalk. While specific inhibitors were not evaluated in this study, the potential to modulate the TME by severing the EV-mediated communication loop suggests that EV-targeted therapies warrant further investigation as a novel treatment approach for canine mammary carcinoma.

This study has several limitations. As the experiments were conducted in vitro, these conditions cannot fully recapitulate the complexity of the in vivo tumor microenvironment. Notably, while EV dosing in this study was standardized by protein concentration (µg/mL), future in vivo investigations should adopt particle-based dosing strategies to ensure greater physiological relevance. Additionally, the use of single tumor (CIPp) and macrophage (DH82) cell lines limits generalizability. Thus, validation in primary cells or multiple tumor models is required. Finally, although the STAT3 axis was implicated as a potential mediator, the precise intracellular signaling cascades driving M2 polarization warrant further detailed molecular characterization.

In conclusion, this study highlights the pivotal role of CIPp-EVs in remodeling the TME through the induction of a pro-tumorigenic feedback loop. These EVs promote the polarization of macrophages into TAMs, which in turn secrete factors that enhance tumor cell migration and EMT, thereby increasing invasive and metastatic potential. Collectively, the findings underscore the significance of the EV–TAM axis in the progression of canine mammary carcinoma. A deeper understanding of this EV–TAM–tumor cell axis may provide a conceptual framework for developing EV- or TAM-targeted therapeutic strategies in canine mammary tumors and other human cancer models that share similar microenvironmental mechanism.

## Supplementary Material

Below is the link to the electronic supplementary material.

Supplementary Material 1 (DOCX 1.08 MB)

Supplementary Material 2 (XLSX 11.6 KB)

## Data Availability

The datasets generated and/or analyzed in the current study are available from the corresponding author upon reasonable request.
