# Supplementary material for: Extracellular vesicles from canine mammary tumor cells promote macrophage M2 polarization and enhance tumor progression
Source: Vet Res Commun. 2026 Mar 30;50(3):243. doi: 10.1007/s11259-026-11179-3 (PMC13035530; doi:10.1007/s11259-026-11179-3)
Supplement: Supplementary file 1 — Supplementary Material 1 (DOCX 1.08 MB) [file 11259_2026_11179_MOESM1_ESM.docx]

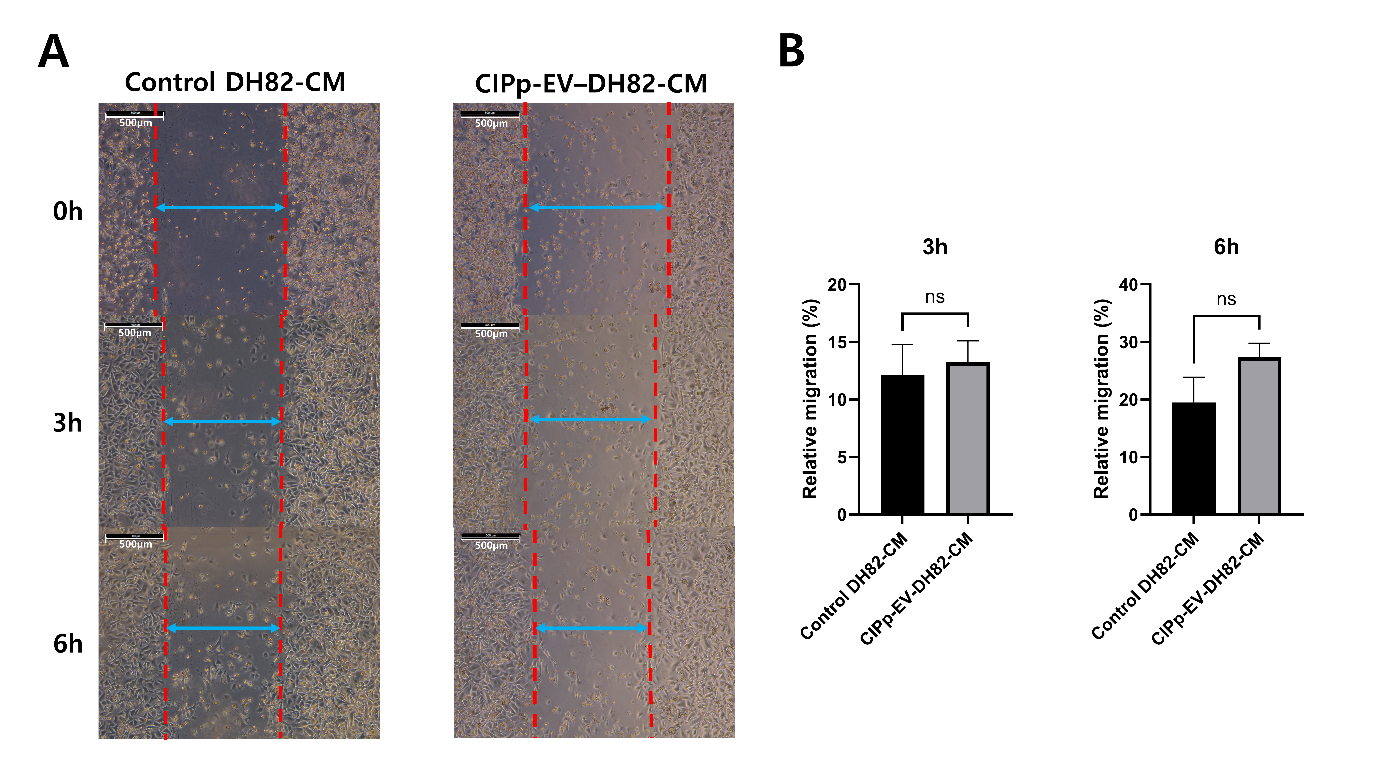


**Fig. S1** CM collected after 36 hours of EV exposure does not enhance CIPp cell migration. CIPp cells were cultured in complete DMEM supplemented with 50% CM derived from DH82 macrophages. CM from untreated macrophages is referred to as Control DH82-CM, whereas CM collected from DH82 macrophages treated with CIPp-EVs for 36 hours is referred to as CIPp-EV–DH82-CM. All experiments were performed in triplicate and repeated as three independent biological experiments. (A) Representative wound closure images at 0, 3, and 6 hours following treatment. No detectable increase in migratory capacity was observed in the CIPp-EV–DH82-CM group compared with control group. Scale bar: 500 μm. (B) Quantification of relative wound closure. Data are presented as mean ± SD (n = 3). ns, not significant versus control.
